# How to Sleep

**Published:** 1883-01

**Authors:** 


### HOW TO SLEEP.

Health and comfort depend very much on attention to matters that to some seem very trival. We have sometimes heard persons complain that they did not sleep well; that they were troubled with horrible dreams, and arose in the morning weary and nervous. Inquiries as to diet, exercise and other essentials of health have often failed to reveal anything that could account for these unfavorable conditions.

It is not well in these cases to limit our investigations to the routine of a day; but we should inquire at what hour the patient goes to bed, what he thinks about usually, and most particular what position he places himself in to invite sleep? If he lies on the back with his hands over his head, there will be a half conscious sense of compression of the chest, with difficult breathing, to relieve which he opens his mouth. The air coming in direct contact with the throat, causes dryness, and then snoring will begin. In the meantime the pressure of the viscera on the large artery, whose course is along the inner portion of the back bone, impedes the circulation of the blood, producing discomfort which manifests itself in horrid dreams. Thus the whole night is passed in a disturbed sleep, and perhaps many nights pass without one of refreshing sleep. The most unwise course under such circumstances would be to resort to the use of opium or any other drug. The ranks of the victims of this unfortunate habit are recruited mainly from such cases as we have described. It is wonderful what control an individual can get over himself if he tries. There is no reason why a person cannot lie upon his side instead of the back, and keep his hands and arms down; then he will not open his mouth; then his throat will not become dry, neither will he snore or have bad dreams. But often he can't help thinking about his business, and his thoughts will run on for hours. This is also a habit that may be broken up. Have the will to put aside your thoughts, and in time you will have the power to do so.

We do not say that there are not other causes that habitually interfere with sound sleep; but we believe there is a remedy for each difficulty which may be found by seeking for it.

Ostrich farming seems to be profitable in South Australia. Mr. W. Malcolm, at Gawler, keeps seventy-four ostriches, most of them reared by himself.

